# Supplementary material for: WordCommentsAnalyzer: A windows software tool for qualitative research
Source: F1000Res. 2018 Sep 4;7:536. Originally published 2018 May 3. [Version 2] doi: 10.12688/f1000research.14819.2 (PMC6137411; doi:10.12688/f1000research.14819.2)
Supplement: Supplementary file 3 [file f1000research-7-17614-s0002.tgz › b2a79496-4ada-47c4-9682-a99ce867836d.zip!/Eight Iranian Health Journals/Archives of Rehabilitation_62.docx]

© Archives of Rehabilitation. This work is licensed under CC BY-NC 4.0 (https://creativecommons.org/licenses/by-nc/4.0/).

Volume & Issue: دوره 5 - شماره 1 و 2

Title: طراحی و معیاریابی آزمون تشخیصی خواندن

http://rehabilitationj.uswr.ac.ir/article-1-136-fa.html

هدف: مقاله حاضر گزارشی از چگونگی طراحی، ساخت و محتوای آزمون تشخیص نارساخوانی است. جمعیت هدف این آزمون کودکان فارسی‌زبانی هستند که قادر به یادگیری خواندن نیستند و چه‌بسا نارساخوان باشند. این آزمون تشخیصی اولین آزمون خواندن معیار برای کودکان فارسی‌زبان است. روش بررسی: روش پژوهش حاضر مقطعی و توصیفی-تحلیلی و چارچوب نظری آن بر مبنای دو آزمون خواندن معتبر انگلیسی به نام‌های «تحلیل مشکلات خواندن دورل» و «تحلیل توانایی خواندن نیل» و محتوای زبانی آن مبتنی‌بر واژگان و متن‌های کتاب فارسی کلاس اول است. بدنه اصلی آزمون مرکب از دو متن داستانی همتا (موازی) است که واژگان و ساختار جملات آن از نظر بسامد، قاعده‌مندی، ساختار هجایی، صرف، نحو و معناشناسی توسط دو متخصص زبان‌شناس و سه معلم کلاس اول به‌دقت کنترل شده است. این آزمون روی 605 دانش‌آموز دختر و پسر پایه اول ابتدایی که به‌صورت تصادفی از مناطق مختلف تهران انتخاب شده‌اند، انجام شده است. یافته‌ها: در این آزمون علاوه بر تعیین صدک‌ها، میانگین‌ها و انحراف معیار دقت و سرعت خواندن، از ضریب همبستگی پیرسون و یومن‌ویتنی نیز استفاده شده است. روایی آزمون از طریق فرم‌های همتا (90% ~) و اعتبار آن به‌طریق اعتبار محتوایی بررسی شده است. نتیجه‌گیری: این آزمون که دارای یک بخش تکمیلی مرکب از زیرآزمون‌های املا، تناظر نویسه-واج، خواندن کلمات بی‌قاعده،خواندن ناکلمه‌ها و دست‌نویس است، می‌تواند در تشخیص نارساخوانی و آسیب‌های مرتبط با خواندن کاربرد داشته باشند.

Title: تأثیر فیلم‌های کمدی در کاهش افسردگی بیماران اسکیزوفرنیک

http://rehabilitationj.uswr.ac.ir/article-1-137-fa.html

مقدمه: این بررسی به‌منظور تأثیر فیلم‌های کمدی در درازمدت، در کاهش افسردگی بیماران اسکیزوفرنیک بستری در بخش‌های توان‌بخشی انجام گرفته است. روش بررسی: 50 نفر از بیماران اسکیزوفرنیک بلوک‌های 5 و 6 بیمارستان روان‌پزشکی رازی به روش نمونه‌گیری آسان انتخاب و به دو گروه 25 نفری مرکب از گروه تجربی و گروه گواه تقسیم شدند. در ابتدا میزان افسردگی انها از طریق آزمون افسردگی بک اندازه‌گیری شد. سپس یک روز در میان، روزی 30 دقیقه به‌مدت دو هفته برای گروه تجربی بخش‌هایی از فیلم‌های کمدی صامت چارلی چاپلین و روآن اتکینسون (مستر بین) به‌نمایش گذاشته شد. برای گروه گواه نیز به‌همان نسبت بخش‌هایی از یک فیلم مستند در مورد پیدایش کره زمین پخش شد. در پایان هفته دوم، میزان افسردگی هر دو گروه به‌وسیله بازآزمون مجدداً اندازه‌گیری شد. یافته‌ها: به‌منظور بررسی معنی‌داری تفاوت میان افسردگی گروه تجربی پیش از مداخله و پس از آن و نیز بررسی گروه گواه، برای نمونه‌های زوجی از آزمون رتبه علامت‌دار ویل کاکسون استفاده شد. برای توصیف داده‌ها، جدول فراوانی و ترسیم نمودارها نیز نرم‌افزار SPSS و Excel مورد استفاده قرار گرفت. طبق این بررسی میان نمره‌های افسردگی گروه تجربی پیش از مداخله و پس از آن تا میزان %95 اطمینان، تفاوت معنی‌دار وجود دارد، ولی میان نمره‌های افسردگی گروه گواه تفاوت معنی‌داری مشاهده نشد. نتیجه‌گیری: فیلم‌های کمدی از نظر آماری سبب کاهش معنی‌دار در افسردگی بیماران اسکیزوفرنیک با اقامت طولانی در بیمارستان روانی می‌شوند.

Title: پذیرش دانش‌آموزان مبتلا به اختلالات شنیداری توسط همسالان در مدارس تلفیقی و غیرتلفیقی (عادی)

http://rehabilitationj.uswr.ac.ir/article-1-138-fa.html

هدف: پژوهش حاضر به‌منظور تعیین میزان پذیرش دانش‌آموزان مبتلا به اختلال شنیداری توسط دانش‌آموزان عادی در مدارس تلفیقی و غیرتلفیقی (عادی) انجام شده است. تحقیقات گوناگون نشان داده‌اند عامل پذیرش دانش‌آموزان استثنایی توسط دانش‌آموزان عادی در موفقیت برنامه‌های تلفیقی یا فراگیرسازی (Inclusion) مؤثر است. روش بررسی: روش این پژوهش، زمینه‌یابی است و برای توصیف میزان پذیرش دانش‌آموزان نیمه‌شنوا از پرسشنامه (Acceptance Scale) استفاده شده است. نمونه‌های تحقیق شامل 323 دانش‌آموز عادی دختر و پسر کلاس‌های سوم، چهارم و پنجم دبستان‌های تهران بودند که همکلاسی مبتلا به اختلال شنوایی داشتند. این نمونه‌ها به‌طور تصادفی از مناطق پنج‌گانه تهران انتخاب گشتند. همچنین این پژوهش برای مقایسه، یک نمونه 65 نفری از دانش‌آموزان همین مقاطع تحصیلی را در مدارس غیرتلفیقی مورد بررسی قرار داد. برای بررسی پایایی یا اعتبار، پایایی تقسیم به نصف اسپرمن–براون +%82 به‌کار گرفته و ضریب آلفا +%77 گزارش شد. در روش آزمون مجدد در دو موقعیت به فاصله سه هفته، ضریب ثبات +%68 بود. در این تحقیق تأثیر جنسیت و نوع مدرسه (تلفیقی، غیرتلفیقی یا عادی) بر میزان پذیرش دانش‌آموزان مبتلا به اختلال شنیداری مورد بررسی قرار گرفت.  یافته‌ها: یافته‌های پژوهش در زمینه عامل جنسیت نشان داد که در مدارس تلفیقی، پذیرش دانش‌آموزان پسر بیشتر از دختران است و در مورد مقایسه میزان پذیرش در مدارس تلفیقی و غیرتلفیقی (عادی)، نتایج میزان پذیرش بالاتر در مدراس تلفیقی را نشان می‌دهند. نتیجه‌گیری: همان‌طورکه نتایج نشان می‌دهند، دانش‌آموزان نیمه‌شنوای مدارس تلفیقی از میزان پذیرش بالاتری توسط همکلاسی‌ها برخوردارند. این برتری در مدارس تلفیقی بیانگر این واقعیت است که تجربه تماس و آشنایی با کودکی که نیازهای ویژه دارد، می‌تواند بر پذیرفته‌شدن این دانش‌آموزان توسط دانش‌آموزان عادی اثرگذار باشد. محدودیت‌های این پژوهش و کم‌بودن پژوهش‌ها در این زمینه، ضرورت پژوهش‌های بیشتر به‌ویژه در زمینه مداخلات مناسب برای افزایش پذیرش را آشکار می‌سازد.

Title: بررسی میزان تأثیر استفاده از گچ‌گیری سریال در رفع خم‌شدگی ثابت مفاصل پروگزیمال انگشتان

http://rehabilitationj.uswr.ac.ir/article-1-139-fa.html

هدف: خم‌شدگی کانترکچر مفصل PIP، پس از شکستگی‌های داخلی مفصلی، خارج مفصلی فالانکس، صدمات تاندونی و… یکی از مشکلات عمده محسوب می‌شود. گچ‌گیری سیلندری از سال 1950 به‌وسیله «دکتر باول براند» در هند به‌عنوان روشی برای رفع بدشکلی‌ها مطرح شد. به‌نظر وی این روش ایجاد استرچ بیشرونده نیست، بلکه رشددادن است. سلول‌های بافت کانترکته‌شده وقتی در حداکثر ext قرار می‌گیرند، به رشد تحریک می‌شوند و به‌طور داخلی مجدداً جهت‌گیری و تغییر می‌کنند. روش بررسی: در این تحقیق 28 انگشت دچار خم‌شدگی کانترکچر که به مرکز دست ایران مراجعه کرده بودند، به‌‌روش Casting تحت درمان قرار گرفتند. در روز مراجعه، میزان T.A.M. 3 خم‌شدگی کانترکچر و خم‌شدگی gap بیماران ارزیابی شد. طی یک ماه هر یک روز در میان، یک‌بار گچ بازمی‌شد و حمام پارافین، ماساژ موبیلیزیشن و تمرینات اکتیو و پاسیو برای مفصل انجام می‌گرفت. سبس، مجددا انگشت در وضعیت بیشتری از اکستنشن قرار می‌گرفت. در انتهای دوره درمان یک‌ماهه ارزیابی مجدد (مشابه موارد ارزیابی اولیه) انجام شد. یافته‌ها: نتایج نشان داد که میانگین T.A.M بیماران افزایش از 88/51 به 130/18 و در مقابل، میانگین خم‌شدگی کانترکچر از 37/59 به 8/14 و خم‌شدگی gap بیماران از 4/37 به 2/37 کاهش داشته است که نشانگر مؤثربودن استفاده از این روش برای درمان خم‌شدگی کانترکچر است. نتیجه‌گیری: یافته‌ها نشان می دهد این روش روش مفیدی برای درمان خم‌شدگی کانترکچر بدون محدودیت در رنج حرکات است.

Title: غربالگری ناشنوایان غیرسندرومی اتوزومال مغلوب برای جهش‌های ژن GJB2

http://rehabilitationj.uswr.ac.ir/article-1-140-fa.html

هدف: کاهش شنوایی 1 نفر از هر 1000 تا 2000 کودک تازه متولد‌شده را تحت تأثیر قرار می‌دهد. بیش از %50 از این موارد را به‌عوامل ژنتیکی نسبت می‌دهند. کاهش شنوایی غیرسندرمی بیش از 70 درصد از موارد ناشنوایی ارثی است که 85 درصد از آن را وراثت جسمی مغلوب دارند و تاکنون بیش از یک‌صد جایگاه (Locus) برای این نوع ناشنوایی برآورد شده است. ژن‌های مختلفی با این ناشنوایی در ارتباط هستند که عمده‌ترین آنها جهش در ژن کانکسین 26 (GJB2) است که در جمعیت‌های مختلف وجود دارد. موتاسیون در این ژن سبب کاهش شنوایی غیرسندرمی اتوزومی مغلوب (Autosomal recessive non syndromic hearing loss) می‌شود. روش بررسی: هدف در این مطالعه غربالگری بیماران برای جهش‌های ژن GJB2 بود. در این تحقیق از تکنیک ARMS/PCR استفاده گردید، نمونه‌هایی که با این روش برای 35 delG هموزیگوت بودند، کنار گذاشته شدند و سپس نمونه‌هایی که هتروزیگوت و یا منفی بودند با روش DHPLC و Direct sequencing بررسی شدند. یافته‌ها: در این تحقیق 76 کروموزوم (38 فرد بیمار) بررسی شد: 32 کروموزوم (%42) در ژن GJB2 جهش داشتند که بالاترین شیوع به جهش 35delG مربوط است. سایر جهش‌ها که در این منطقه دیده شد شامل W24X R127H, R32H, -3170G>A بودند. همچنین پلی‌مورفیسم V153I در سه فرد بیمار (8/3) تشخیص داده شد. نتیچه‌گیری: این آمار با سایر آمار جهان مشابهت چندانی ندارد و بیانگر وجود احتمالی ژن‌ها و لوکوس‌های دیگر دخیل در این منطقه است.

Title: شناسایی اختلالات رفتاری در کودکان دارای پدر معتاد

http://rehabilitationj.uswr.ac.ir/article-1-141-fa.html

هدف: اعتیاد والدین (والد) می‎تواند منجر به پیدایش یا افزایش میزان اختلالات رفتاری در کودکان شود. پژوهش حاضر به بررسی میزان اختلالات رفتاری در کودکان دارای پدر معتاد و مقایسه آن با میزان این اختلالات در کودکان دارای پدر غیرمعتاد اختصاص دارد. روش‎ بررسی: اختلالات رفتاری در 250 دانش‎آموز متشکل از 125 دانش‌آموز با پدر معتاد و 125 دانش‌آموز با پدر غیرمعتاد با استفاده از پرسشنامه اختلالات رفتاری راتر -ویژه معلمان- مورد بررسی قرار گرفت.  یافته‌ها: یافته‌های پژوهش نشان داد که میزان اختلالات رفتاری در کودکان دارای پدرمعتاد بیشتر از کودکان دارای پدر غیرمعتاد بود. میزان اختلالات رفتاری در پسران بیشتر از دختران بود. میزان تحصیلات پدر با میزان اختلالات رفتاری فرزندان رابطه داشت. با افزایش تحصیلات پدر میزان اختلالات رفتاری فرزندان کاهش می‌یافت. میزان غیبت از مدرسه و تعداد دفعات ارجاع به مشاور مدرسه در کودکان دارای پدر معتاد نسبت به کودکان دارای پدر غیرمعتاد بیشتر بود. بین اختلالات رفتاری و پیشرفت تحصیلی همبستگی منفی معنادار وجود داشت. نتیجه‎گیری: بین اعتیاد پدر و اختلالات رفتاری در کودکان رابطه وجود دارد.

Title: انطباق و هنجاریابی نسخه بازبینی‌شده آزمون مهارت‌های بینایی-حرکتی

http://rehabilitationj.uswr.ac.ir/article-1-142-fa.html

هدف: انطباق، رواسازی و هنجاریابی آزمون مهارت‌های بینایی-حرکتی با توجه به اهمیت یکپارچگی بینایی-حرکتی در اکثر فعالیت‌های روزمره زندگی و همچنین دو عملکرد اساسی خواندن و نوشتن انجام شد. روش بررسی: این عمل روی نمونه‌ای با حجم 1281 نفر در فاصله سنی (2 سال و 11 ماه) تا (13 سال و 11 ماه) شهر تهران که با روش نمونه‌برداری چندمرحله‌ای انتخاب شدند، اجرا گردید. این آزمون از 23 طرح هندسی تشکیل شده است، هر طرح از طریق یک معیار مشخص و دقیق ارزیابی می‌شود و از طریق موارد غلط (ضعف) و صحیح (قوت) نمره‌گذاری می‌شود. برای انطباق و هنجاریابی این آزمون، ابتدا کتابچه راهنما و کتابچه معیار نمره‌گذاری به زبان فارسی ترجمه شد. با اجرای مراحل تجربی و نهایی و تجزیه و تحلیل داده‌ها براساس مدل کلاسیک اعتبار، فرم نهایی آزمون شکل گرفت. ضریب همسانی درونی خرده‌آزمون‌ها (طبقه‌بندی‌ها) با استفاده از فرمول آلفای کرونباخ و ثبات زمانی خرده‌آزمون‌ها (طبقه‌بندی‌ها) با استفاده از روش آزمون و باز‌آزمون به‌دست آمد. روایی محتوایی، ملاکی و سازه خرده‌آزمون‌ها (طبقه‌بندی‌ها) با استفاده از روش‌های مناسب مورد بررسی قرار گرفت. یافته‌ها: نتایج به‌دست‌آمده در زمینه به‌دست‌آمده در زمینه اعتبار و روایی آزمون، اطمینان کافی برای استفاده از این ابزار را برای ارزیابی مهارت‌های بینایی-حرکتی کودکان شهر تهران به‌دست می‌دهد. نتیجه‌گیری: براساس نتایج حاصل‌شده از این آزمون به‌عنوان ابزاری جهت مشخص‌کردن اختلال در هماهنگی چشم و دست، شناسایی کودکان مبتلا به اختلال در مهارت های بینایی-حرکتی و مستندکردن پیشرفت کودکان در زمینه مهارت‌های حرکتی ظریف به‌ویژه مهارت‌های بینایی-حرکتی در کودکان 14-3 سال استفاده نمود.

Title: مقایسه توانایی ادراک فضاشناختی دهانی در کودکان طبیعی 6 و 7 ساله

http://rehabilitationj.uswr.ac.ir/article-1-143-fa.html

هدف: پژوهش حاضر به شناسایی معیارتشخیص شکل از طریق دهانی در کودکان طبیعی 6 و 7 ساله شمال و جنوب شهر تهران و همچنین برآورد میانگین مدت زمان تشخیص شکل (سرعت پاسخ‌دهی) از طریق حس‌های دهانی توسط این کودکان می‌پردازد تا بتوان از آن در ارزیابی و درمان اختلالات حس‌های دهانی و اختلالات تولید گفتار در کودکان سنین مدرسه استفاده کرد. روش بررسی: این پژوهش روی 200 کودک 6 و 7 ساله طبیعی شمال و جنوب شهر تهران انجام شد که به‌طورمساوی از دو گروه سنی فوق و از دو گروه جنسی انتخاب شدند. هیچ یک از کودکان مبتلا به اختلالات فکی-دندانی، اختلالات آناتومیک اندام‌های گویایی، آپراکسی دهانی، مشکلات بینایی و اختلالات اعصاب مغزی کرانیال نبودند. آزمون مورد استفاده از پژوهش حاضر شامل 20 شی بود که به‌صورت تصادفی داخل دهان افراد قرار داده می‌شد تا کودک شکل آن را از بین سه گزینه تصویری که در مقابل داشت نشان دهد و سپس درستی و نادرستی پاسخ ها به‌همراه مدت زمان صرف‌شده از ارائه هر شیء تا ارائه اولین پاسخ توسط فرد محاسبه شد. یافته‌ها: میانگین امتیازات در آزمون حسی تشخیص شکل در کودکان 6 ساله 17/34 و در کودکان 7 ساله 17/59 از 20 است. تفاوت میانگین این دو گروه سنی معنی‌دار نیست (0/31=P). همچنین میانگین مدت زمان تشخیص شکل در 6 ساله‌ها 2/67 ثانیه و در 7 ساله‌ها 2/82 ثانیه است و تفاوت میانگـین این دو گروه معنی‌دار نیست (0/11=P). علاوه‌براین کودکان شمال شهر نسبت به کودکان جنوب شهر تهران سرعت پاسخدهی کمــتری دارند (0/000=P). همچنین دخترها به نسبت پسرها تنها از نظر زمان به‌طور معنی‌داری سریع‌تر پاسخ می‌دهند (0/043=P). نتیجه‌گیری: براساس این پژوهش، میان کودکان 6 ساله و 7 ساله، در توانایی درک فضا شناختی دهانی تفاوت قابل ملاحظه‌ای مشاهده نمی‌شود. همچنین سرعت پاسخدهی در کودکان جنوب شهر سریع‌تر از کودکان شمال شهر است. نظر به اهمیت این حس در تولید گفتار، هنجاریابی آن در سنین مختلف توصیه می‎گردد.

Title: مقایسه اثر بانداژکشی و مچ‌بند نئوپرنی بر حس وضعیت مفصلی و درد بیماران مبتلا به پیچ‌خوردگی درجه 1و2

http://rehabilitationj.uswr.ac.ir/article-1-144-fa.html

هدف: کاربرد روش‌های درمانی مؤثر در توان‌بخشی افرادی که مبتلا یپچ خوردگی درجه 1و2 مچ پا هستند، در کاهش مدت درمان و افزایش بازتوانی می‌تواند گام مؤثری تلقی گردد. هدف از این پژوهش تعیین تأثیر مچ‌بند نئوپرنی و بانداژکشی روی حس وضعیت مفصلی و درد بیماران پیچ‌خورده با درجه 1و2 و مقایسه میزان تأثیر آنها بود. در یک مطالعه شبه‌تجربی 30 نفر (14زن-16مرد) از افراد پیچ‌خورده پای درجه 1و2 با سنین 52-16 سال مورد آزمانیش قرار گرفتند درد بیماران بر اساس VAS قبل از آزمایش می‌بایست 2 سانتی‌متر از 10 سانتی‌متر باشد. روش بررسی: پای بیماران در حالت نشسته به‌طور فعال در زاویه بین 0 تا 15 درجه پلانتار فلکشن قرار می‌گرفت که همان زاویه هدف بود و بعد از 5 ثانیه مکث با چشمان بسته و دست‌به‌سینه این زاویه 3 مرتبه بازسازی می‌شد. تفاضل میانگین سه دفعه بعد با زاویه هدف برای مقایسه استفاده می‌شد. برای تعیین میزان درد بیماران از معیار سنجش دیداری VAS استفاده شد که در حالت بدون درد این میزان 0 سانتی‌متر و در حالت درد شدید به 10 سانتی‌متر نشان داده می‌شد و مراحل بالا قبل و بعد از 20 دقیقه تکراری گردید. یک هفته بعد مداخله بعدی انجام می‌شد. یافته‌ها: با استفاده از تی زوج‌شده، علامت‌های رتبه‌دار ویل‌کگسون، ضریب همبستگی پیرسون داده‌ها، مورد تجزیه و تحلیل قرار گرفت و نشان داد که میانگین خطای زاویه‌ای قبل و بعد از مچ‌بند نئوپرنی تفاوت معنی‌داری دارد (0/034=P) و مچ‌بند حس وضعیت مفصلی را بهبود بخشیده است، ولی بانداژ اثری روی حس وضعیت مفصلی ندارد (0/539=P) در مورد درد بیماران هم بانداژ و هم مچ‌بند درد را به‌طور معنی‌داری کاهش دادند که این اثر در مورد مچ‌بند بیشتر بوده است (0/000=P). نتیجه‌گیری: در این مطالعه میزان اثر مچ‌بند نئوپرنی در حس وضعیت مفصلی و درد بیماران مبتلا بیشتر از بانداژکشی بوده است؛ بنابراین، جهت تعیین تأثیر بانداژکشی پیشنهاد می‌شود که انواع دیگر بانداژ مورد بررسی قرار گیرند.

Title: تأثیر بروموکریپتین، فلاکستین ونورتریپتیلین در کاهش علائم منفی اسکیزوفرنیا

http://rehabilitationj.uswr.ac.ir/article-1-145-fa.html

هدف: علائم منفی در اسکیزوفرنیا یکی از عوامل مهم بازدارنده در فرایندهای مرتبط با توان‌بخشی این دسته از بیماران محسوب می‌گردد. داروهای کمکی و آموزش مهارت‌های اجتماعی می‌توانند جهت تقلیل شدت این دسته از علائم به‌کار روند. در این پژوهش نقش بروموکریپتین، فلاکستین و نورتریپتیلین را در کاهش علائم منفی مورد بررسی قرار می‌دهیم. در این بین نقش نورتریپتیلین برای اولین بار است که مورد مطالعه اینچنینی قرار می‌گیرد. روش بررسی: یک‌صد بیمار اسکیزوفرنیک بستری در مرکز روان‌پزشکی رازی به‌طور تصادفی انتخاب شدند و پس از تقسیم به چهار گروه مساوی، در یک آزمون دوسوکور و کنترل‌شده به‌ترتیب با بروموکریپتین 5/2 میلی‌گرم، فلاکستین 20 میلی‌گرم، نورتریپتیلین 25 میلی‌گرم و بالاخره پلاسبو، به‌اضافه رژیم درمانی جاری‌شان (شامل آنتی‌سایکوتیک‌های تیپیکال) تحت درمان قرار گرفتند. پس از سه هفته دوز داروها به دو برابر افزایش یافت و این روند به‌مدت سه هفته دیگر ادامه یافت و سپس داروها قطع گردید. وجود و شدت علائم منفی در ابتدا و قبل از آغاز درمان و سپس در انتهای هفته سوم و بالاخره در انتهای هفته ششم با استفاده از مقیاس SANS کنترل و ثبت گردید. در طی این مدت (6 هفته) یک بیمار از گروه فلاکستین به‌دلیل عدم تمایل و بیمار دیگری از گروه پلاسبو به‌دلیل فوت در اثر بیماری جسمانی از مطالعه حذف شدند. صرف‌نظر از این دو مورد، هیچ‌گونه عارضه جانبی خاصی که موجب ناراحتی بیماران با قطع دارو گردد، مشاهده نشد. پس از استخراج اطلاعات به‌دست‌آمده و با استفاده از روش محاسبات آماری Z و chi-square و X2-test ارتباط بین متغیرها سنجیده گردید. یافته‌ها: علائم منفی در چهار گروه مذکور تحت تأثیر پلاسبو، بروموکریپتین، فلاکستین، و نوروتریپتیلین به‌ترتیب در %37/5، %44، %62/5 و %80 از بیماران دچار تقلیل گردید. این کاهش در اکثریت قریب به‌اتفاق موارد محدود به %20 از سطح پایه باقی ماند. فقط نورتریپتیلین در سه مورد و بروموکریپتین در یک مورد به میزان %40 علائم منفی را کاهش دادند. از حیث شدت علائم پاسخ‌دهنده به درمان، تفاوتی میان انواع شدید و غیرشدید مشاهده نگردید. همچنین تقلیل مورد بحث علائم منفی به‌طور غیروابسته به یکدیگر و به‌صورت متفرق صورت گرفته بود. نتیجه‌گیری: هر سه داروی بروموکریپتین، فلاکستین و نورتریپتیلین موجب تقلیل علائم منفی بیماران اسکیزوفرنیک گردیدند. از این بین در مقایسه با پلاسبو، نورتریپتیلین مؤثرترین (P<0/005) و بروموکریپتین ضعیف‌ترین (P<0/75) بودند و فلاکستین نیز در حد واسط بین این دو قرارداشت (P<0/1). اگرچه بین نورتریپتیلین و فلاکستین تفاوت معنی‌داری ملاحظه نشد (P<0/25)، اما نورتریپتیلین به‌طور مشخص مؤثرتر از بروموکریپتین (P<0/01) بود. این امر در مورد مقایسه فلاکستین با بروموکریپتین صادق نبود (P<0/25).

Title: جنبه‌های روان‌شناختی درد و چگونگی ادراک آن در کودکان

http://rehabilitationj.uswr.ac.ir/article-1-146-fa.html

درد یک تجربه و احساس همگانی است. گویاترین تعریف، درد را چنین توصیف می‌کند: احساس آسیب فردی و خصوصی. این تعریف، درد را از دیدگاه ذهنی فرد توصیف می‌کند و به‌طور ضمنی به این امر اشاره دارد که فرد دردمند کاملاً بر احساس خود واقف است. تعاریف دیگر تکیه بر سودمندی درد دارند. به ‌عنوان مثال، درد نشانه ای از وجود یک محرک آسیب‌زا در آسیب‌دیدگی‌های موضعی است که الگویی از پاسخ‌ها را برای حفاظت فرد فعال می‌سازد. پزشکان باتجربه، غالباً دریافته‌اند که درد پیامدی از اختلال عاطفی است که در اقلیت ثابتی از بیماران مشاهده می‌شود. فراوان اتفاق افتاده است که پزشک به بیماران دردمند خود گفته است دردی وجود ندارد؛ زیرا شواهدی دال بر اختلال عضوی به‌چشم نمی‌خورد. اکثر پزشکان با پیامدهای اجتنابی و ناخوشایند این قبیل خطاها آشنا هستند. در این صورت بهتر است با تجربه بیمار که خود آن را «درد» می‌نامد، موافقت نموده و در همه موارد در پی سازوکار جسمانی سبب‌ساز نباشیم. ثابت شده است که عوامل روان‌شناختی نیز می‌تواند سبب درد باشد که در این صورت آن را «روان‌زاد» می‌نامند. این اصطلاح به‌معنی آن است که در این موارد علل درد، تماماً یا غالباً ریشه روانی دارد و از سوی دیگر، درد عضوی به آن‌گونه درد می‌گویند که عوامل سبب‌ساز آن جسمی‌ باشد. در این تحقیق رویکردهای نظری، دیدگاه‌های روان‌شناختی، دیدگاه‌های روان تحلیلگری و دیدگاه‌های روانی-اجتماعی و نیز بیان ویژگی‌های شخصیتی به‌همراه توصیف درد و ارتباط آنها مورد بررسی قرار داده شده است. همچنین چگونگی ادراک در کودکان و در جنسیت دختر و پسر با استفاده از یافته‌های پژوهش مورد بررسی قرار گرفته است.

Title: یافته‌های پرتونگاری مغز در آتاکسی تلانژکتازی

http://rehabilitationj.uswr.ac.ir/article-1-147-fa.html

آتاکسی تلانژکتازی، یک بیماری ارثی است که به روش اتوزوم مغلوب منتقل می‌شود. علائم این بیماری به‌صورت تلانژکتازی‌های پوستی و ملتحمه، آتاکسی مخچه‌ای و کمبودهای ایمنی است. شیوع بالایی از تومورهای بدخیم وجود دارد که بیشتر به‌صورت لنفوم است. آتروفی مخچه بارزترین آنومالی بوده که در پرتونگاری از سیستم اعصاب مشاهده می‌شود. خون‌ریزی مغزی نیز به‌ندرت ایجاد می‌شود. بیماری که معرفی می‌گردد یک پسر 7 ساله بوده که به‌علت عفونت‌های مکرر ریوی بستری گردید. در معاینه، راه‌رفتن بیمار آتاکسیک بود و واکنش‌های وتری عمقی در اندام‌های تحتانی کاهش داشت. در ملتحمه بیمار، تلانژکتازی وجود داشت و تکلم وی نامفهوم و گنگ بود. در پرتونگاری از مغز به روش رزونانـس مگنتیک (MRI) آتروفی مخچه و تغییرات هیپرانتنسیته در ماده سفید مغز مشاهده گردید که در نواحی پس‌سری بارزتر و مطرح‌کننده لکودیستروفی بود. این تغییرات در ماده سفید مغز، در بیماری آتاکسی تلانژکتازی قبلاً گزارش نشده است.
